# Superantigen Encoding Genes in *Staphylococcus aureus* Isolated from Lesional Skin, Non-Lesional Skin, and Nares of Patients with Atopic Dermatitis

**DOI:** 10.2340/actadv.v104.34882

**Published:** 2024-06-11

**Authors:** Natalia RATUSZNIK, Sofie Marie EDSLEV, Marc STEGGER, Bo SÖDERQUIST

**Affiliations:** 1 School of Medical Sciences, Faculty of Medicine and Health, Örebro University, Örebro, Sweden; 2 Department of Bacteria, Parasites & Fungi, Statens Serum Institut, Copenhagen, Denmark; 3 Antimicrobial Resistance and Infectious Diseases Laboratory, Harry Butler Institute, Murdoch University, Perth, Australia

**Keywords:** atopic dermatitis, *staphylococcus aureus*, superantigens, enterotoxins, TSST-1

## Abstract

Patients with atopic dermatitis (AD) are more likely than healthy individuals to harbour *Staphylococcus aureus* on their skin. Superantigens (SAgs) produced by specific *S. aureus* strains may contribute to AD-associated skin inflammation. The present study compared the prevalence and types of SAg-encoding genes between *S. aureus* isolated from patients with AD and from controls, and within the AD group between isolates from different sampling sites (lesional skin, non-lesional skin, and nares)*.* This retrospective case-control study extracted data from 2 previous studies that examined *S. aureus* using whole-genome sequencing. The 138 *S. aureus* isolates obtained from 71 AD patients contained 349 SAg-encoding genes; 22 (6.3%) were found in isolates from nares (0.4 ± 0.6 genes per isolate), 99 (28.4%) in isolates from non-lesional skin (3.7 ± 3.9), and 228 (65.3%) in isolates from lesional skin (4.2 ± 4.5). *S. aureus* (*n* = 101) from the control group contained 594 SAg-encoding genes (5.9 ± 4.2). Of the *S. aureus* isolated from lesional AD skin, 69% carried at least 1 gene encoding SAg compared with 33% of AD nasal isolates. SAg could be a factor in the pathogenesis of a subset of AD patients.

SIGNIFICANCE

Patients with atopic dermatitis are more likely than healthy individuals to carry *Staphylococcus aureus* on their skin. *Staphylococcus aureus* superantigens may be responsible for inflammation associated with atopic dermatitis. We investigated the presence of *Staphylococcus aureus* superantigen-encoding genes in *Staphylococcus aureus* isolated from atopic dermatitis patients and from controls. Almost all *Staphylococcus aureus* superantigen-encoding genes were identified among *Staphylococcus aureus* from both patients and controls, indicating heterogeneous *Staphylococcus aureus* superantigen gene profiles. However, *Staphylococcus aureus* superantigen-encoding genes were seen in two-thirds of *Staphylococcus aureus* isolates from lesional skin compared with only one-third of atopic dermatitis nasal isolates, suggesting that *Staphylococcus aureus* superantigens may be a factor in the pathogenesis of atopic dermatitis.

Atopic dermatitis (AD) is a common chronic skin disease affecting up to 20% of children and 10% of adults (1, 2), characterized by T-cell-dependent inflammation and impaired skin barrier function. Its aetiology is multifactorial, including genetic factors such as loss-of-function mutations in the gene encoding filaggrin, immune dysregulation, and colonization or infection with *Staphylococcus aureus*. In comparison with healthy individuals, AD patients are more likely to harbour *S. aureus* on their skin and also exhibit a significantly greater abundance of it (3–6). Increased skin colonization with *S. aureus*, and reduced alpha-diversity, has been linked to more severe AD disease, possibly due to the expression of various virulence factors such as phenol-soluble modulins, clumping factor B, alpha-haemolysin, and enterotoxins with the potential to act as superantigens (SAgs) (7). SAgs can bind to major histocompatibility complex class II molecules on antigen-presenting cells and T-cell receptors, leading to unspecific T-cell activation even in the absence of antigenic peptide presentation, and hence to cytokine release (3, 7). Thus, inflammation in AD skin can be maintained and exacerbated if SAgs are expressed, as they are potent immune system activators.

Three main categories of SAgs have been identified in *S. aureus*: staphylococcal enterotoxins (SEs), toxic shock syndrome toxin-1 (TSST-1), and staphylococcal enterotoxin-like (SE-*l*) SAgs. SEs were originally named for their ability to induce emesis when ingested, but as new SEs were discovered, the nomenclature was revised. For example, TSST-1 (originally named SEF before being renamed in 1983) and SE-*l* SAgs either lack or have not been tested for emetic activity, but still possess T-cell superantigenic properties (8).

The prevalence of SAg-encoding genes appears to be higher in *S. aureus* isolated from AD patients who are non-responsive to treatments involving steroids and tacrolimus, compared with the general AD population (9, 10). As methicillin-resistant *S. aureus* is a global issue, targeting SAgs could be an alternative to antibiotics for therapy-resistant AD (11). As AD patients report drastically reduced quality of life, it is essential to map the SAg gene profile in *S. aureus* from AD skin, as this could lead to novel treatments in the future (12). This study therefore used whole-genome sequencing (WGS) data to provide new insights into the potential significance of carriage of SE, TSST-1, and SE-*l-*encoding genes in *S. aureus* isolated from AD patients.

The aims of this study were: (*i*) to compare the prevalence and types of SAg-encoding genes between *S. aureus* isolated from AD patients and from healthy controls, (*ii*) to make the same comparison within the AD group between different sample sites (nares, lesional skin, non-lesional skin), and (*iii*) to evaluate the frequency of SAg genes among *S. aureus* isolated from lesional AD skin.

## MATERIALS AND METHODS

### Study design

This retrospective case-control study was based on 2 prior studies involving isolation and sequencing of *S. aureus*. The first study (*Study 1*) was conducted in Denmark, and examined the antibiotic resistance of *S. aureus* from 71 AD patients (13). The second study (*Study 2*) was conducted in Sweden, and explored the presence of *S. aureus* in the nares of 101 patients scheduled for hip or knee replacement surgery (17). Genome sequence data for *Studies 1* and *2* were available from the European Nucleotide Archive under accession numbers PRJEB18560 and PRJEB33164, respectively.

### Atopic dermatitis patients and controls

*Study 1:* During 2013–2015, 33 male and 38 female patients aged 18–77 (mean: 36.7) were enrolled from the Department of Dermatology, Bispebjerg University Hospital, Denmark. The inclusion criteria were being aged ≥ 18 years, having AD according to the UK Working Party’s Diagnostic Criteria for Atopic Dermatitis, and harbouring *S. aureus* in at least 1 of 3 sites: lesional skin, non-lesional skin, and anterior nares (13,14). These patients constituted the AD patient group of the present study.

*Study 2*: The controls in Study 2 comprised 2 cohorts of anonymized patients from 1992 (46 patients) and 2017 (55 patients) who were scheduled for elective arthroplasty surgery at the Department of Orthopedic Surgery, Örebro University Hospital, Sweden, and who provided isolates of *S. aureus* from the anterior nares (15). Healthy skin is essential for undergoing arthroplasty, as it reduces the risk of postoperative infections; these patients were therefore suitable controls for the present study, as it can be assumed that their skin was in good condition (16).

### Staphylococcus aureus isolate collection, DNA extraction, WGS, and genetic typing

*Study 1:* Bacterial swabs were taken from lesional skin, non-lesional skin (from the volar forearm or the antecubital crease), and the anterior nares using ESwabs (Copan, Brescia, Italy) (17). *S. aureus* was identified by plating on chromID *S. aureus* plates (bioMérieux, Marcy-l’Étoile, France). One isolate was taken from each sample for further investigation. A total of 54 *S. aureus* isolates were obtained from lesional skin, 27 from non-lesional skin, and 57 from the anterior nares. DNA was extracted from the isolates using a Qiagen DNeasy Blood and Tissue Kit (Qiagen, Hilden, Germany). WGS was conducted using a Nextera XT DNA Library Preparation Kit and a MiSeq sequencer (Illumina Inc, San Diego, CA, USA). In the present study, the clonal complexes (CCs) were determined from the sequence types provided in *Study 1* using PHYLOViZ Online (18).

*Study 2*: Isolates of *S. aureus* were collected from the anterior nares with Rayon Swabs (Copan, Brescia, Italy) in 1992 and with ESwabs (Copan) in 2017. DNA was extracted from isolates using the QIAsymphony DSP Virus/Pathogen kit (Qiagen), and WGS was carried out with the Nextera DNA Library Preparation XT kit (Illumina) on a MiSeq (Illumina). eBURST was used to assign CC from the sequence types (15).

### Identification of genes of interest

*SAg-encoding genes.* Data were drawn from the Virulence Factor Database (http:/www.mgc.ac.cn/VFs). DNA sequences of the full dataset were downloaded in February 2023 and imported into version 2023.0.4 of Geneious Prime (Biomatters Ltd, Auckland, New Zealand). All VFs previously identified in *S. aureus* named either *enterotoxin*, *TSST-1*, or *enterotoxin-like* were selected, extracted, and aligned using MUSCLE. Based on the between-VF dissimilarity, if the same VFs identified in different strains of *S. aureus* had over 90% sequence similarity, it was deemed sufficient to include only 1 of these VFs in subsequent detection analyses. Analyses of the nucleotide alignments were also used to assess the sequence diversity between different VFs and hence to determine whether some VFs were similar enough to generate false-positive hits in BLAST-based analyses (19).

*SAg-encoding genes in S. aureus isolates***.** *S. aureus* genomic data from *Studies 1* and *2* were analysed using version 2023.0.4 of Geneious Prime. A batch search using MegaBLAST was run over a custom database of the *S. aureus* draft genomes. For detection of genes, the criteria for considering a BLAST hit as positive were set to an E-value of at least 0.001 and query coverage and pairwise identity of at least 90% (21).

The prevalence rates of SAg-encoding genes were calculated by dividing the number of positive hits for each gene by the total number of *S. aureus* isolates in each respective subgroup. The χ^2^ test and Fisher’s exact test were used when suitable (dichotomous outcome: any SAg-encoding gene vs no SAg-encoding gene) to compare gene carriage in the *S. aureus* isolates from the controls and from the different sample sites in AD patients. The Kruskal–Wallis H test was used to compare the number of SAg-encoding genes between isolates from nares, non-lesional skin, and lesional skin. *P*-values below 0.05 were considered statistically significant but were consequently Bonferroni adjusted in the sub-analyses. All tests were performed in version 28 of IBM SPSS Statistics (IBM Corp, Armonk, NY, USA).

### Ethical considerations

This was an extended analysis of publicly available WGS data for *S. aureus* isolates from studies that had received ethical approval. *Study 1* was approved by the local ethics committee (project number 1-2014-039) and the Danish Data Protection Agency (project number 01767 BBH-2012-019) and all participants provided informed consent (13). *Study 2* was approved by the Regional Ethical Review Board of Uppsala, Sweden (ref: 2016/151, 2016/151/1, and 2016/151/2), and the collection of nasal isolates in 1992 was approved by a Chair’s decision of the Regional Ethical Committee in Örebro, Sweden (15).

## RESULTS

### S. aureus from atopic dermatitis patients compared with controls

**Fig. 1:**
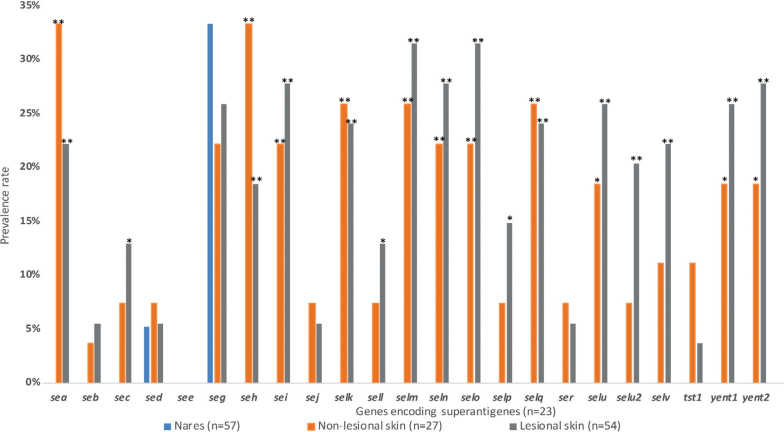
**Prevalence rates of genes encoding superantigens for each sampling site in patients with atopic dermatitis.** No statistically significant differences were found between isolates from non-lesional skin and isolates from lesional skin, thus all indicated *p*-values refer to comparisons between nares and non-lesional skin (χ^2^ test for *seg*, Fisher’s exact test for all other gene types) and between nares and lesional skin (Fisher’s exact test for *seb*, *sed*, *sej*, *sell*, *selp*, *ser*, and *tst*1, χ^2^ test for all other gene types). *P*-values below 0.017 were considered statistically significant according to the Bonferroni multiple comparison test, as 3 tests were conducted for each gene. *p < 0.017, **p < 0.001.

We studied the presence of 23 SAg-encoding genes (*sea*-*see*, *seg-sej*, *selk-selq*, *ser*, *selu*, *selu2*, *selv*, *tst1*, *yent1*, and *yent2*) in *S. aureus* isolates collected from AD patients and controls with presumed healthy skin. The WGS data for 138 *S. aureus* isolates from 71 AD patients identified 349 SAg-encoding genes, of which 22 (6.3%) were found in isolates from the nares (0.4 ± 0.6 genes per isolate), 99 (28.4%) from the non-lesional skin (3.7 ± 3.9 genes per isolate), and 228 (65.3%) from the lesional skin (4.2 ± 4.5 genes per isolate). WGS data for the 101 *S. aureus* isolates from the nares of the controls contained 594 SAg-encoding genes (5.9 ± 4.2 genes per isolate). All studied genes and their prevalence rates are shown in **Table I** and **Fig. 1**. The statistical analyses comparing the isolates obtained from controls with the isolates from AD patients are shown in Table I, while the comparisons between different sampling sites within the AD group are illustrated in Fig. 1.

**Table I:** Number and types of superantigen-encoding genes in *Staphylococcus aureus* isolated from the control group and from patients with atopic dermatitis (AD). Pairwise comparison for each gene between *S. aureus* isolates from the control group and from each sampling site of the AD patients

Superantigen-encoding gene	*S. aureus* isolates from the nares of the control group, *n* (%); 101 (100)	*S. aureus* isolates from the nares of AD patients, *n* (%); 57 (100)	*S. aureus* isolates from non-lesional skin of AD patients, *n* (%); 27 (100)	*S. aureus* isolates from lesional skin of AD patients, *n* (%); 54 (100)
*sea*	20 (19.8)	0 **^a^	9 (33.3)^a^	12 (22.2)^a^
*seb*	4 (4.0)	0^b^	1 (3.7)^b^	3 (5.5)^b^
*sec*	11 (10.9)	0 *^b^	2 (7.4)^b^	7 (13.0)^a^
*sed*	4 (4.0)	3 (5.3)^b^	2 (7.4)^b^	3 (5.5)^b^
*see*	0	0	0	0
*seg*	60 (59.4)	19 (33.3)*^a^	6 (22.2)**^a^	14 (26.0)**^a^
*seh*	5 (5.0)	0^b^	9 (33.3)**^b^	10 (18.5)*^a^
*sei*	52 (51.5)	0**^a^	6 (22.2)*^a^	15 (27.8)*^a^
*sej*	4 (4.0)	0^b^	2 (7.4)^b^	3 (5.5)^b^
*selk*	2 (2.0)	0^b^	7 (26.0)**^b^	13 (24.1)**^a^
*sell*	11 (10.9)	0*^b^	2 (7.4)^b^	7 (13.0)^a^
*selm*	61 (60.4)	0**^a^	7 (26.0)*^a^	17 (31.5)**^a^
*seln*	47 (46.5)	0 **^a^	6 (22.2)^a^	15 (27.8)^a^
*selo*	59 (58.4)	0**^a^	6 (22.2)**^a^	17 (31.5)*^a^
*selp*	6 (5.9)	0^b^	2 (7.4)^b^	8 (14.8)^b^
*selq*	2 (2.0)	0^b^	7 (26.0)**^b^	13 (24.1)**^a^
*ser*	4 (4.0)	0^b^	2 (7.4)^b^	3 (5.5)^b^
*selu*	48 (47.5)	0**^a^	5 (18.5)*^a^	14 (26.0)*^a^
*selu2*	25 (24.8)	0**^a^	2 (7.4)^a^	11 (20.4)^a^
*selv*	32 (31.7)	0**^a^	3 (11.1)^a^	12 (22.2)^a^
*tst1*	27 (26.8)	0**^a^	3 (11.1)^a^	2 (3.7)**^a^
*yent1*	55 (54.5)	0**^a^	5 (18. 5)**^a^	14 (26.0)**^a^
*yent2*	55 (54.5)	0**^a^	5 (18.5)**^a^	15 (27.8)*^a^

*P*-values below 0.017 were considered statistically significant according to the Bonferroni multiple comparison test, as 3 tests were conducted for each gene.

* *p* < 0.017,

** *p* < 0.001,

a χ^2^ test,

b Fisher’s exact test.

The prevalence of the genes *seh*, *selk*, and *selq* was significantly higher in *S. aureus* isolates from both lesional and non-lesional skin of the AD patients, compared with controls (Table I). The *selk* and *selq* genes were always present together, and this pair was observed in 84% of *seh*-positive *S. aureus* isolates. The enterotoxin gene cluster (EGC) consisting of *seg*, *sei*, *selm*, *seln, selo*, and *selu* was present in 13 (24.7%) *S. aureus* isolates from lesional skin, 4 (14.8%) isolates from non-lesional skin, 0 nasal isolates from AD patients, and 31 (30.7%) isolates from controls. No isolates from either controls or AD patients carried *seb* and *tst1* simultaneously.

### S. aureus from AD patients

A total of 37 (68.5%) *S. aureus* isolates from lesional skin, 19 (70.0%) *S. aureus* isolates from non-lesional skin, and 19 (33.0%) *S. aureus* isolates from the nares carried at least one SAg-encoding gene. Among controls, 81 (80.2%) *S. aureus* isolates from the nares carried at least one SAg-encoding gene.

**Fig. 2:**
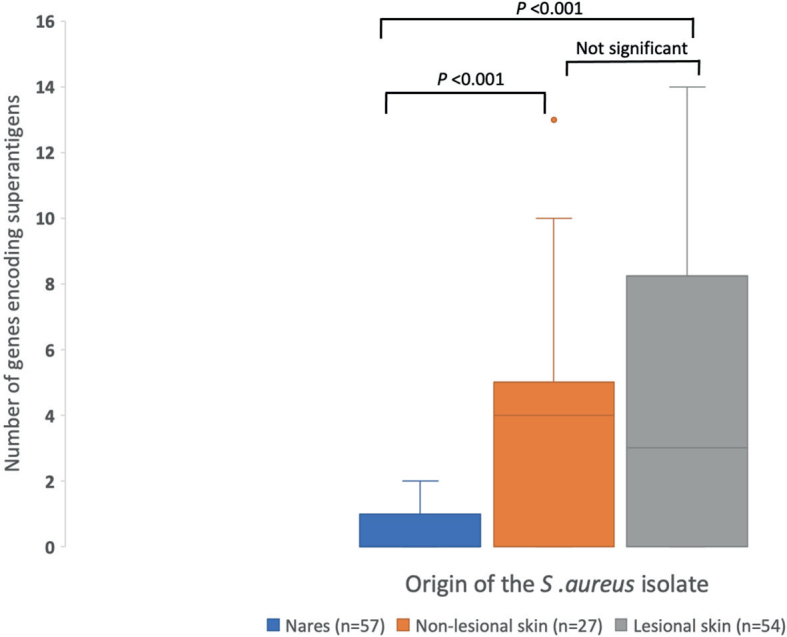
**Range of genes encoding superantigens in *Staphylococcus aureus* isolates from each sampling site in patients with atopic dermatitis (nares, non-lesional skin, and lesional skin).** *P*-values were calculated with the Kruskal–Wallis H test followed by pairwise comparisons conducted with Dunn’s test; the significance values were adjusted by the Bonferroni correction for multiple tests.

*S. aureus* isolates from AD patients demonstrated 0–14 SAg-encoding genes, with the ranges depending on the sample origin. The median value for gene carriage was 0 in the nares, 4 in non-lesional skin, and 3 in lesional skin (**Fig. 2**).

**Fig. 3:**
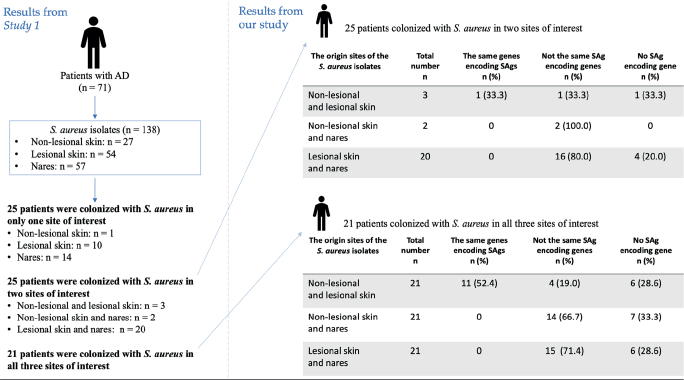
**Comparative analysis of genes encoding superantigens (SAgs) in *Staphylococcus aureus* from different origin sites in atopic dermatitis patients colonized by *S. aureus* at two or three sample sites.** *Study 1* refers to Edslev et al. (13).

Among the 24 AD patients who had *S. aureus* on both lesional and non-lesional skin, 12 (50.0%) had isolates with identical SAg-encoding genes, 5 (20.8%) had different SAg-encoding genes depending on the sample origin, and 7 (29.2%) had no SAg-encoding genes. None of the 43 patients colonized with *S. aureus* in both nares and lesional or non-lesional skin shared the same genes encoding SAgs in their respective *S. aureus* isolates (**Fig. 3**).

### Clonal complexes

Seventeen different CCs were found among the *S. aureus* isolated from the 71 AD patients, with CC1, CC15, CC30, and CC45 being the most common types. Nineteen different *S. aureus* CCs were identified in the 101 controls, with CC30, CC45, CC15, and CC25 being the most frequent types (**Table II**). None of the CC15 isolates carried any SAg-encoding genes in either group, while CC5 isolates always carried at least one SAg gene. All CC45 isolates detected from AD patients carried ≥ 1 SAg-encoding gene (**Table III**). CC1, which was common among AD patients, did not carry any unique SAg gene.

**Table II:** Prevalence of the most common *Staphylococcus aureus* clonal complexes (CCs) in the control group and in patients with atopic dermatitis (AD)

*S. aureus* CC type	*S. aureus* from the nares, control group (*n* = 101) *n* (%)	*S. aureus* from the nares, AD patients (*n* = 57) *n* (%)	*S. aureus* from non-lesional skin, AD patients(*n* = 27) *n* (%)	*S. aureus* from lesional skin, AD patients (*n* = 54) *n* (%)
**CC1**	3 (3)	13 (23)	8 (30)	11 (20)
**CC5**	5 (5)	5 (9)	1 (4)	4 (7)
**CC7**	1 (1)	3 (5)	2 (7)	2 (4)
**CC8**	4 (4)	2 (3)	2 (7)	4 (7)
**CC15**	8 (8)	11 (19)	5 (19)	11 (20)
**CC22**	5 (5)	–	–	–
**CC25**	6 (6)	–	–	–
**CC30**	28 (28)	5 (9)	3 (11)	3 (6)
**CC45**	23 (23)	9 (16)	2 (7)	8 (15)
Other*	18 (18)	9 (16)	4 (15)	11 (20)

* Other: either ≤4 S. aureus isolates of a distinct CC type across all isolates, or not part of any recognized CC.

**Table III:** Relationship between *Staphylococcus aureus* clonal complexes (CCs) and the carriage of genes encoding superantigens (SAgs)

*S. aureus* CC type	*S. aureus* from the control group, 0 SAg genes (*n* = 20) *n* (%)	*S. aureus* from the control group, ≥1 SAg genes (*n* = 81) *n* (%)	*S. aureus* from AD patients,0 SAg genes (*n* = 62) *n* (%)	*S. aureus* from AD patients, ≥ 1 SAg genes (*n* = 76) *n* (%)
**CC1**	2 (10)	1 (1)	13 (21)	19 (25)
**CC5**	0	5 (6)	0	10 (13)
**CC7**	0	1 (1)	3 (5)	4 (5)
**CC8**	0	4 (5)	7 (11)	1 (1)
**CC15**	8 (40)	0	27 (43)	0
**CC22**	0	5 (6)	–	–
**CC25**	0	6 (7)	–	–
**CC30**	0	28 (35)	1 (2)	10 (13)
**CC45**	3 (15)	20 (25)	0	19 (25)
Other*	7 (35)	11 (14)	11 (18)	13 (17)

* Other: either ≤4 S. aureus isolates of a distinct CC type across all isolates, or not part of any recognized CC.

## DISCUSSION

The overall prevalence of SAg-encoding genes was higher in *S. aureus* isolates from the controls than in isolates from AD patients. We identified 22 of the 23 examined SAg-encoding genes among *S. aureus* isolates from both groups, demonstrating heterogeneous SAg-encoding gene profiles. No significant difference in SAg carriage was observed between isolates from lesional and non-lesional skin, and *sed* and *seg* were the only gene types identified among isolates from the nares of AD patients. At least one SAg-encoding gene was detected among 69% of *S. aureus* isolates from lesional skin.

The higher prevalence of SAg-encoding genes in the control group may be attributed to the age difference; patients awaiting knee or hip replacement surgery likely have a higher average age than the AD patients from *Study 1*, whose mean age was 36.7 years. A previous study showed that the distribution of the *seb* gene in *S. aureus* is age-related, with this gene being detected in 46.5% of *S. aureus* isolates from infants, 58.3% from children, and 71.4% from adults (22). The population structure of *S. aureus* may be different among different age groups and as such present different SAg profiles as detected in our collection. On the other hand, it could also be lineage-dependent; that is, an effect of the lineages that were present, because, for example, all CC45 and CC30 isolates carried at least one SAg gene.

It is important to emphasize that only the presence of SAg-encoding genes was investigated, and not their expression. *S. aureus* in AD skin seems to express SAgs to a greater extent than isolates from the skin of non-AD individuals (6, 10, 23). This could be due to the quorum-sensing system in *S. aureus*, where a higher bacterial density is related to increased virulence gene expression (24). Data suggests a significantly higher *S. aureus* prevalence across both lesional and non-lesional skin areas as well as within the anterior nares of AD patients in comparison with controls (7, 25). Furthermore, the absolute abundance of *S. aureus* is particularly pronounced in lesional skin relative to non-lesional areas within the AD group. This implies a greater likelihood of encountering expression of the SAg-encoding genes within isolates obtained from AD patients, particularly those from lesional sites, in contrast to isolates from controls. Hence, despite the high prevalence of SAg-encoding genes in controls, their expression may be limited. Further studies examining both gene carriage and expression would be informative.

Although > 90% of the patients colonized with *S. aureus* in multiple sample sites had only 1 type of CC, the isolates did not possess the same SAg-encoding genes. This may seem counterintuitive, as bacteria with the same CC are typically genetically similar and thus have similar characteristics. However, like other VFs such as antibiotic resistance genes, SAg-encoding genes are usually located on the mobile genetic elements (MGE), which allows significant genetic variation due to MGE acquisition and loss (26). It is possible that the possession of SAg-encoding genes could shift due to horizontal gene transfer among bacteria, even within 1 host. However, we cannot infer the directionality of such events, and both a gain and a loss of SAg-encoding genes in *S. aureus* could contribute to the observed differences.

The remarkable discrepancy in the number of SAg-encoding genes in *S. aureus* isolates between nasal and skin samples from AD patients could be attributed to a more challenging microenvironment on the skin, especially non-lesional skin, as proposed recently (9). The nasal mucosa is enriched with natural adhesin ligands, and is a typical colonization niche for *S. aureus* (27), while skin may be less hospitable for bacterial survival. As a result, more virulent *S. aureus* isolates could be selected on the skin, as secretion of SAgs can disrupt the skin barrier and thus promote bacterial survival. It is possible that the lack of SAg-encoding genes may also be beneficial for persistent *S. aureus* colonization of the nasal mucosa, as less-pathogenic *S. aureus* can more easily evade the immune system.

In addition, there was a notable high proportion of nasal isolates from the control group carrying SAg genes compared with the nasal samples from the AD patients; 14 genes were significantly more prevalent in the control group compared with the AD nares.

Our results are consistent with those reported by several other studies. For example, *S. aureus* from lesional AD skin in the present study exhibited a near-identical carriage rate of the EGC (24.7% vs 24%) compared with a study analysing SAg profile in *S. aureus* from patients with steroid-resistant AD (10). This set of genes was highlighted, as it is commonly found in *S. aureus* isolated from AD skin, and a correlation has been reported between EGC carriage and increased severity of AD (9, 28). Moreover, the *see* gene was not detected in any of the *S. aureus* isolates from either AD patients or controls, in line with results from at least 6 other studies (29). None of the present isolates carried *seb* and *tst1* simultaneously, and it is commonly assumed that the proteins SEB and TSST-1 are not co-produced in *S. aureus* (30).

Our study has some limitations. We did not evaluate correlations between the prevalence or types of SAg-encoding genes and the severity of AD. In addition, it was not possible to adjust our findings for age, sex, and comorbidities, due to the anonymous nature of the control group and a strict data agreement that did not allow us to obtain this information on the AD patients. Future studies along these lines should ensure that none of the controls have a history of AD, other inflammatory skin diseases or atopies; moreover, the control group should be age-matched to the patient group, and control samples should be taken from the skin as well as the nares. The participants were enrolled from 2 Scandinavian countries (Denmark and Sweden) during different time periods (the control group in 1992 and 2017 and the AD patients in 2013–2015), and so the outcomes cannot be generalized. The time gap might also have affected our results, due to potential changes in the SAg profile of *S. aureus* over time (28).

The strengths of this study include the comparatively large study cohort; most studies in this field include fewer than 50 AD patients. In addition, unlike many prior studies, we included a control group with individuals who were expected to have healthy skin due to this being a requirement for undergoing joint replacement surgery (16, 29). Finally, to our knowledge, no previous study has investigated as many SAg-encoding genes in *S. aureus* isolates from AD patients as in the present study; many previous reports have focused solely on the classical SE genes *sea*, *seb*, *sec*, and *tst-1* (31).

### Conclusion

Our findings suggest that SAg-encoding genes may play a role in the pathogenesis of a subset of AD patients, as 69% of *S. aureus* isolated from lesional skin carried at least 1 SAg-encoding gene, which could potentially be linked to local inflammation. Further investigations are needed to ascertain the significance of these virulence factors, as the prevalence was higher among *S. aureus* collected from presumed healthy individuals than from AD patients.
